# Ensemble learning enhances the precision of preliminary detection of primary hepatocellular carcinoma based on serological and demographic indices

**DOI:** 10.3389/fonc.2024.1397505

**Published:** 2024-06-17

**Authors:** Mengxia Wang, Bo Zhuang, Shian Yu, Gang Li

**Affiliations:** ^1^ School of Medicine, Shaoxing University, Shaoxing, Zhejiang, China; ^2^ Department of Hepatobiliary Surgery, The Affliated Jinhua Hospital of Zhejiang University School of Medicine, Jinhua, Zhejiang, China; ^3^ College of Mathematical Medicine, Zhejiang Normal University, Jinhua, China

**Keywords:** primary hepatocellular carcinoma (PHC), ensemble learning, serological and demographic indices, preliminary detection, machine learning

## Abstract

Primary hepatocellular carcinoma (PHC) is associated with high rates of morbidity and malignancy in China and throughout the world. In clinical practice, a combination of ultrasound and alpha-fetoprotein (AFP) measurement is frequently employed for initial screening. However, the accuracy of this approach often falls short of the desired standard. Consequently, this study aimed to investigate the enhancement of precision of preliminary detection of PHC by ensemble learning techniques. To achieve this, 712 patients with PHC and 1887 healthy controls were enrolled for the assessment of four ensemble learning methods, namely, Random Forest (RF), LightGBM, Xgboost, and Catboost. A total of eleven characteristics, comprising nine serological indices and two demographic indices, were selected from the participants for use in detecting PHC. The findings identified an optimal feature subset consisting of eight features, namely AFP, albumin (ALB), alanine aminotransferase (ALT), platelets (PLT), age, alkaline phosphatase (ALP), hemoglobin (Hb), and body mass index (BMI), that achieved the highest classification accuracy of 96.62%. This emphasizes the importance of the collective use of these features in PHC diagnosis. In conclusion, the results provide evidence that the integration of serological and demographic indices together with ensemble learning models, can contribute to the precision of preliminary diagnosis of PHC.

## 1 Introduction

PHC includes some of the most common and fatal malignant tumors. The most common form of PHC is hepatocellular carcinoma (HCC), followed by intrahepatic cholangiocarcinoma (ICC) and combined hepatocellular-cholangiocarcinoma (cHCC-CCA). Although the exact pathogenesis of PHC is not yet fully understood, it is clear that its etiology is diverse. Infection with hepatitis B virus (HBV) is the predominant cause of HCC in Asia (excluding Japan) and parts of Africa, while infection with hepatitis C virus (HCV) is more common in Europe, the United States, and Japan (1). Chronic hepatitis typically progresses to liver fibrosis, cirrhosis, and eventually liver cancer, a chain reaction described as the “liver cancer trilogy.” Globally, PHC ranks sixth in the diagnosis of new cancer cases per year (about 906 000 new cases) and third in annual deaths (approximately 830 000 deaths) (2). It is predicted that the global incidence of PHC will continue to rise, reaching an estimated incidence of 14.08 cases per 100 000 by 2030. Projections suggest a steady increase in PHC incidence until 2030, highlighting a continuous rise without intervention (3).

The increasing incidence of PHC and the generally poor survival outcomes of patients with PHC are both concerning. Approximately two-thirds of PHC patients are diagnosed at an intermediate to advanced stage and survive for less than one year (4). Preliminary detection during physical examinations and the early identification of PHC can facilitate comprehensive evaluations and prompt interventions, thereby offering a range of therapeutic choices. Approaches such as radical surgical resection and diverse adjuvant therapies can significantly improve the 5-year survival rate to 70−75% (5), crucial for improving patient prognosis.

Liver ultrasonography (US) combined with the measurement of AFP levels is commonly used as an initial screening method in clinical settings but its diagnostic accuracy is limited. Many PHC patients also have underlying chronic liver diseases, and the ultrasound imaging of cirrhosis and cirrhosis-associated PHC are strikingly similar. Thus, a sole dependence on ultrasound for PHC diagnosis yields a sensitivity ranging from 60% to 80% (6). Additionally, imaging results can vary due to various factors, such as the quality of the equipment, the expertise of the physician, and the condition of the patient, limiting its reliability. In contrast, hematological measurements provide objective values that can be easily analyzed in clinical settings. If biomarkers can achieve high accuracy in PHC diagnosis, their application in primary screening could be invaluable. It has been suggested that abnormal increases in the levels of blood markers precede abnormalities visible on imaging tests (7), highlighting their potential for preliminary tumor detection. Therefore, the effective improvement of the rates of PHC detection using a combination of biomarkers and ensemble learning has attracted significant attention in recent years.

In the present era, the integration of artificial intelligence (AI) technology into the medical domain has become increasingly prevalent. In response, a plethora of scholars have fervently dedicated themselves to the realm of deep learning, meticulously delving into medical image segmentation techniques to yield remarkable outcomes through the proposal of efficient and practical models (8–11). Within this plethora of research endeavors, precise segmentation models tailored specifically for liver US and CT images abound (12–14). These highly effective segmentation techniques have played an instrumental role in providing substantial support for clinical surgical treatments, aiding in intervention decision-making, and enhancing postoperative evaluations for HCC (15–17). Moreover, the application of AI in the medical domain extends beyond imaging. With the continuous accumulation of clinical big data, a multitude of diagnostic models for PHC, leveraging hematological indicators, are rapidly emerging, further underscoring AI’s transformative potential in healthcare. Among them, a model proposed by Johnson et al. (18) was validated on international multi-center data and was found to correctly classify 91% of patients. However, the incidence of PHC in China differs from that in Europe and the United States, and an evaluation of the performance of this model in the Chinese population by Huang et al. (19) showed reduced diagnostic efficacy. Therefore, the development of a model that is better suited to the Chinese PHC patients’ population would be an effective solution to this problem.

In this study, we analyzed and modeled patient data using ensemble learning techniques. These 11 features were modeled, and a high classification accuracy was obtained. Diagnosing PHC is inherently intricate, and this study endeavors to aid physicians in predicting and diagnosing this condition by constructing a model utilizing commonly available clinical data. In essence, our contributions are as follows:

1. Given that physical examination is widely available and convenient, serological and demographic indicators were chosen as the cornerstones for building predictive models.
2. Fusion of advanced ensemble learning and commonly used indicators of physical examination is proposed to build a PHC primary screening model, while the highest classification accuracy and its corresponding optimal feature subset are obtained through feature selection and importance ranking.

The subsequent sections of the paper are organized as follows: “Challenges and objectives” describes the challenges we faced during the research process and our objectives. The “Materials and methods” section delineates the experimental framework, elucidating details on the dataset, preprocessing techniques, evaluation metrics, and implementation strategies. “Results and Discussion” Section highlights the results of the empirical study and discusses the critical observations and findings. Lastly, the “Conclusion” section encapsulates our contributions and provides a concise summary to conclude the paper.

## 2 Challenges and objectives

In the process of constructing an ensemble learning model for PHC risk prediction, we aim to enhance the model’s predictive capability by collecting a large amount of clinical data. However, this process also encounters significant challenges, which are accompanied by clear objectives. The primary challenge lies in the complexity and diversity of medical data. Such data often contain a large number of missing and abnormal values, which can directly impact the effectiveness of model training. Therefore, we must conduct thorough data cleaning and preprocessing to ensure the quality and integrity of the data. Furthermore, medical data is characterized by numerous and complex features, which may have redundancy and correlation among them, increasing the complexity of the model and potentially affecting its predictive performance. As a result, we need to apply appropriate feature selection methods to reduce the feature dimension, extract the most predictive information, and thereby enhance the accuracy and efficiency of the model.

The core objective of building this PHC prediction model is to improve prediction accuracy. To achieve this goal, we will take a series of measures, including carefully selecting the most predictive features, optimizing the model structure and algorithm, and adopting suitable evaluation metrics to continuously test and enhance the model’s performance. Ultimately, we hope that this model can become a valuable assistant for clinicians, helping them more accurately assess medical examiners’ PHC risk, provide personalized treatment plans, and assist in evaluating treatment outcomes.

## 3 Materials and methods

### 3.1 Study participants

**Figure 1:**
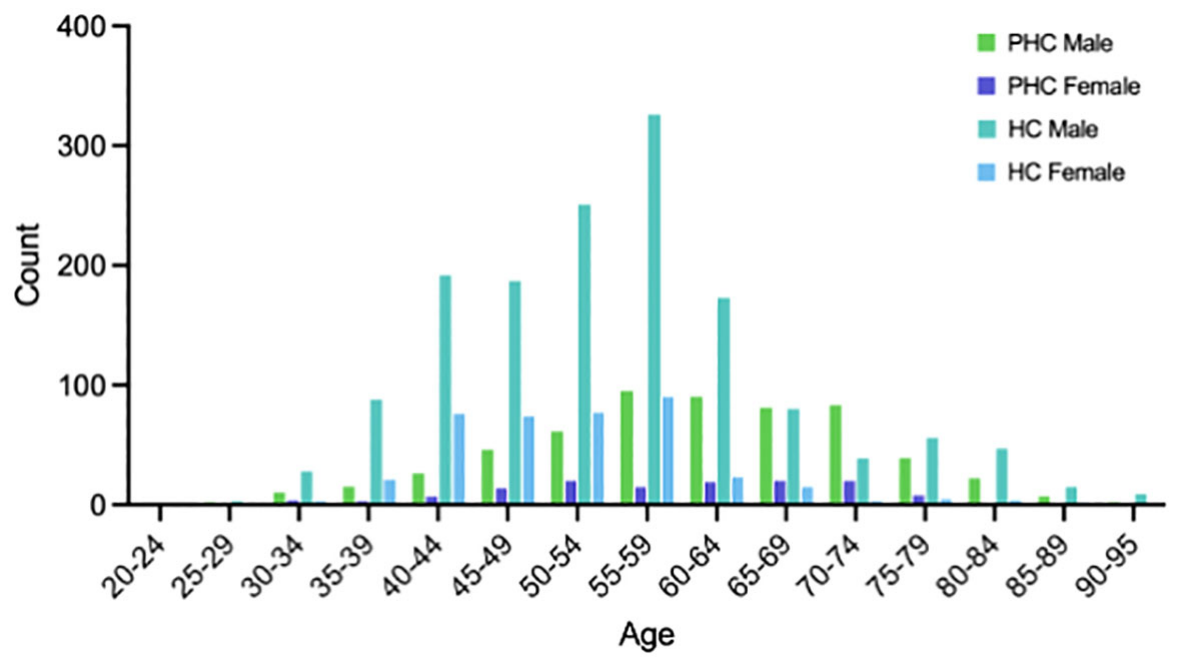
Age and sex distribution of the PHC and HC group.

The clinical data of 712 patients diagnosed with PHC and confirmed by pathological examination in the Department of Hepatobiliary and Pancreatic Surgery of Jinhua Central Hospital between January 2017 and February 2023 were used as the PHC group. The average age in the group was 61.65 ± 12.13 years and the patients included 579 males and 133 females. Additionally, data from the Health Management Center of Jinhua Central Hospital from June 2019 to August 2022, consisting of 1887 physical examinations, were used for the healthy control (HC) group. The average age of the 1887 members of the HC group was 54.11 ± 11.46, and the group included 1494 males and 393 females. The age and sex distributions of both the PHC and HC group are depicted in **Figure 1**. Notably, as shown in **Figure 1**, there was a significantly higher number of male patients compared to females in the PHC group, and most patients were over 50 years old. The incidence rate of PHC among young and middle-aged individuals was lower than that in the older population. The original contributions presented in the study are included in the **Supplementary Material**.

### 3.2 Inclusion and exclusion criteria

Inclusion criteria: PHC group (1): Pathologic diagnosis of PHC after examination of specimens obtained after hepatectomy or puncture biopsy; (2) Complete clinical data. HC group: (1) Age older than18 years old; (2) Test results of various indices in the normal range; (3) No family history of tumors or history of the diagnosis and treatment of liver-related diseases; (4) Abdominal ultrasound imaging data without evidence of diseases of the liver and biliary system; (5) No abnormalities in the results of routine blood tests; (6) No abnormalities in the test results of liver function and renal function; (7) Overall diagnosis of health.

Exclusion criteria: (1) Incomplete general information and imperfect data of related tests and inspections in the medical case; (2) Combinations with other malignant tumors or metastatic hepatic carcinoma (MHC); (3) Combination with severe cardiovascular and cerebrovascular disease or contraindications to anesthesia; (4) Death in the perioperative period.

### 3.3 Observation indices and evaluation criteria

The observational indicators and criteria for evaluation were divided into two main categories: (1) Demographic indices: age and BMI; (2) Serological markers: AFP, white blood cell (WBC), Hb, PLT, ALB, glucose (GLu), total bilirubin (TBil), ALT, and ALP.

### 3.4 Data extraction

Patient data were retrieved from the e-case system of the data center. The data included the initial results of blood, biochemistry, and tumor marker tests obtained from the patients with PHC hospital admission. Similarly, the corresponding test outcomes of the HC group, along with their general clinical information, were extracted from the health management center.

### 3.5 Ensemble learning model

The core concept of ensemble learning lies in “group intelligence,” in which collective decision-making is considered superior to individual choices. In the realm of machine learning, the achievement of satisfactory results using a single model is often challenging, as the predictive performances of different models tend to vary and are thus termed weak classifiers. Increasing the predictive capability of machine models involves an important method, namely, the use of ensembles of machine learning models. The use of ensemble learning allows the amalgamation of multiple weak classifiers into more robust and comprehensive strong classifiers using specific strategies. The present study focused primarily on four integration learning models, namely, RF, LightGBM, Xgboost, and Catboost.

RF trains multiple decision trees through the random extraction of features and samples. There are thus minimal or no correlations among the trees. The final prediction results are obtained by the summation of the predictions of each tree. This methodology is rooted in bootstrap aggregating (Bagging) (20). The RF algorithm specifically gauges the importance of each feature by evaluating its contribution to the individual decision trees. These contributions are assessed by calculations of the Gini index before and after the branching of features on the parse node (21). While RF significantly mitigates data overfitting, thus enhancing training accuracy, it requires significant computational resources as multiple models require simultaneous training.

Gradient Boosting Decision Tree (GBDT) is a boosting method that relies on the concept of residual reduction. It constructs a new model aimed at reducing residuals (negative gradient), thereby diminishing bias. Its predictive capability significantly surpasses that of a single model (22). LightGBM, Xgboost, and Catboost are examples and efficient implementations of GBDT.

LightGBM is an open-source gradient-boosting framework based on decision trees that was introduced by Microsoft. It is able to handle large volumes of data and can enhance the computational efficiency of GBDT. LightGBM utilizes a histogram-based algorithm and a leaf-growing strategy with a maximum depth limit to accelerate training and minimize memory usage. This assists in the reduction of storage and computational costs (23).

Xgboost addresses the issue of model overfitting by the introduction of second-order derivatives and regularization together with increasing the speed and efficiency of the model. It refines predictions iteratively by the continuous splitting of features in growing the tree, and progressively fits residuals from earlier prediction models. By the training’s conclusion, K trees are generated. In each tree, specific features of the sample are associated with specific leaf nodes and scores, with the scores of individual trees summed to derive the predicted value for that sample (24).

Catboost, a recently developed algorithm for the gradient boosting of decision trees, highlights competitive performance, rivaling the leading machine learning algorithms (25). It has two major advantages. First, it can handle categorical data during the training, rather than the preprocessing, stage, thus avoiding the need to encode classification features for model development from raw data. Second, it uses a novel method for calculating leaf values during the selection of tree structure, thus helping to prevent overfitting, a common problem that reduces the generalizability of machine learning models (26).

**Figure 2:**
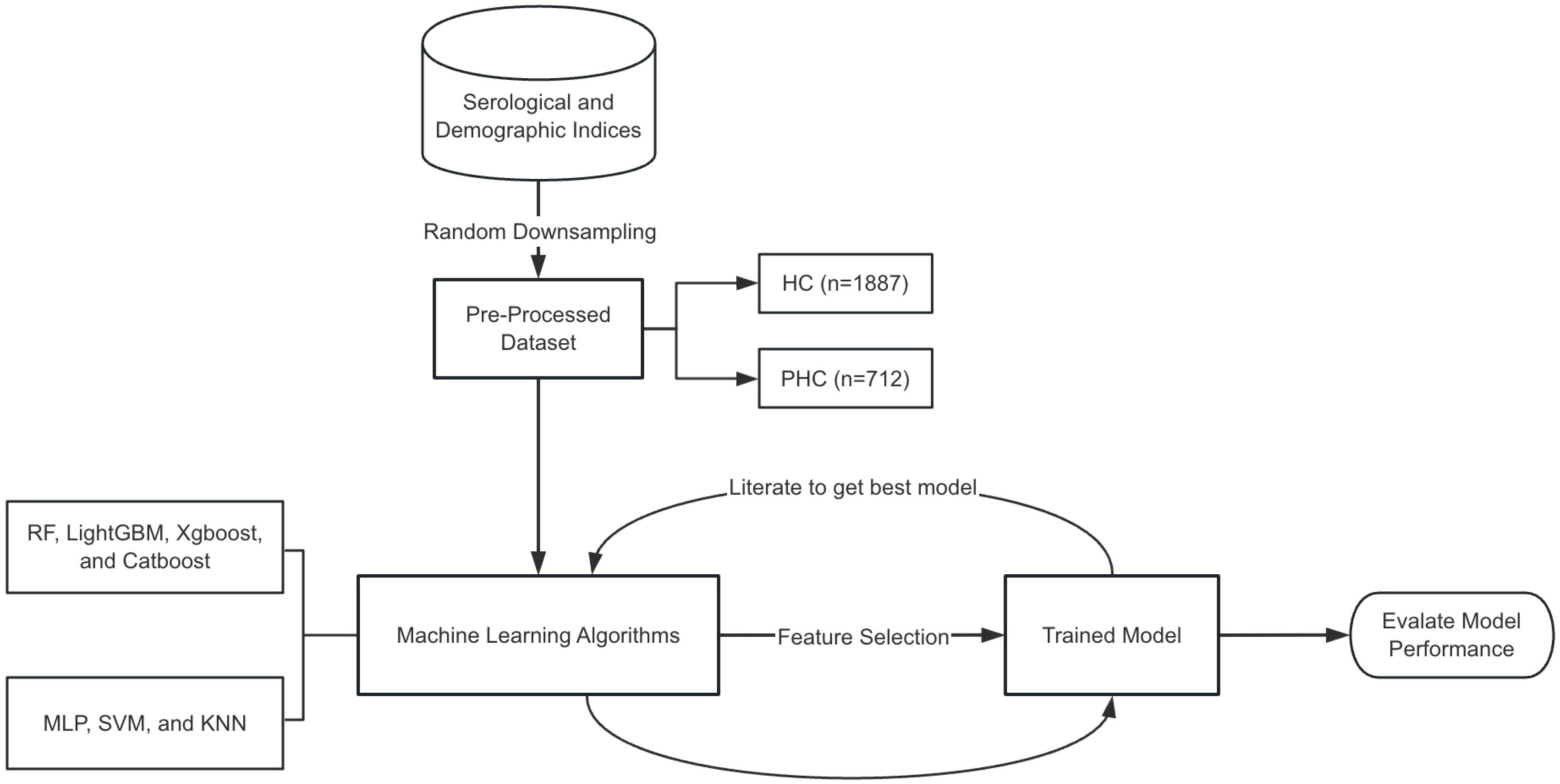
The process of model establishment.

As shown in **Figure 2**, constructing an ensemble learning model entails several key steps. Initially, the collected clinical data undergo preprocessing, which involves procedures such as data normalization and random sampling classification. This preprocessing ensures that the data is appropriately formatted for subsequent machine learning algorithms. Then, the ensemble learning algorithms (RF, LightGBM, Xgboost, and Catboost) are selected, and three classical machine learning methods - Multi-Layer Perceptron (MLP), Support Vector Machine (SVM), and K-Nearest Neighbors (KNN) - are introduced as references for baseline comparative evaluation. Next, feature selection techniques are utilized to further optimize the model’s performance, and finally, the optimized model is evaluated. The hyperparameters of each model used in the study are included in the **Supplementary Material**.

The model developed in this study was a dichotomous model that was evaluated using the confusion matrix (CM) method. The main evaluation metrics used in this study include accuracy, precision, recall, and F1-score, and their formulas are respectively:


$$Accuracy=\frac{TP+TN}{TP+FP+FN+TN}$$

$$Precision=\frac{TP}{TP+FP}$$

$$Recall=\frac{TP}{TP+FN}$$

$$F1-score=\frac{2\;*\;Perscision\;*\;Recall}{Perscision+Recall}$$

### 3.6 Feature ranking and the optimal feature subset

The Recursive Feature Elimination (RFE) algorithm was applied in Catboost, the top-performing classification model, Catboost, for feature selection. The RFE is designed to prevent overfitting and increase model generalization by iterative training of the model and the removal of weaker features until the desired number is reached. Here, RFE was used to evaluate and rank the importance of each feature variable. The optimal subset of features that could distinguish between patients with PHC and healthy individuals was examined using a classification task. Starting with a single feature, further features were added incrementally according to their ranking positions. This iterative process was continued until all features were included in the classifier. This allowed the identification of the optimal subset of features that yielded the highest classification accuracy.

### 3.7 Statistical analysis

The 11 characteristics contained in the above datasets were analyzed statistically using MATLAB 2022b software, with comparisons between the two groups analyzed using one-way analysis of variance (ANOVA) for multiple sets of data, using a test level of α = 0. 05 to assess statistically significant (P<0.05) independent risk factors.

## 4 Result

**Figure 3:**
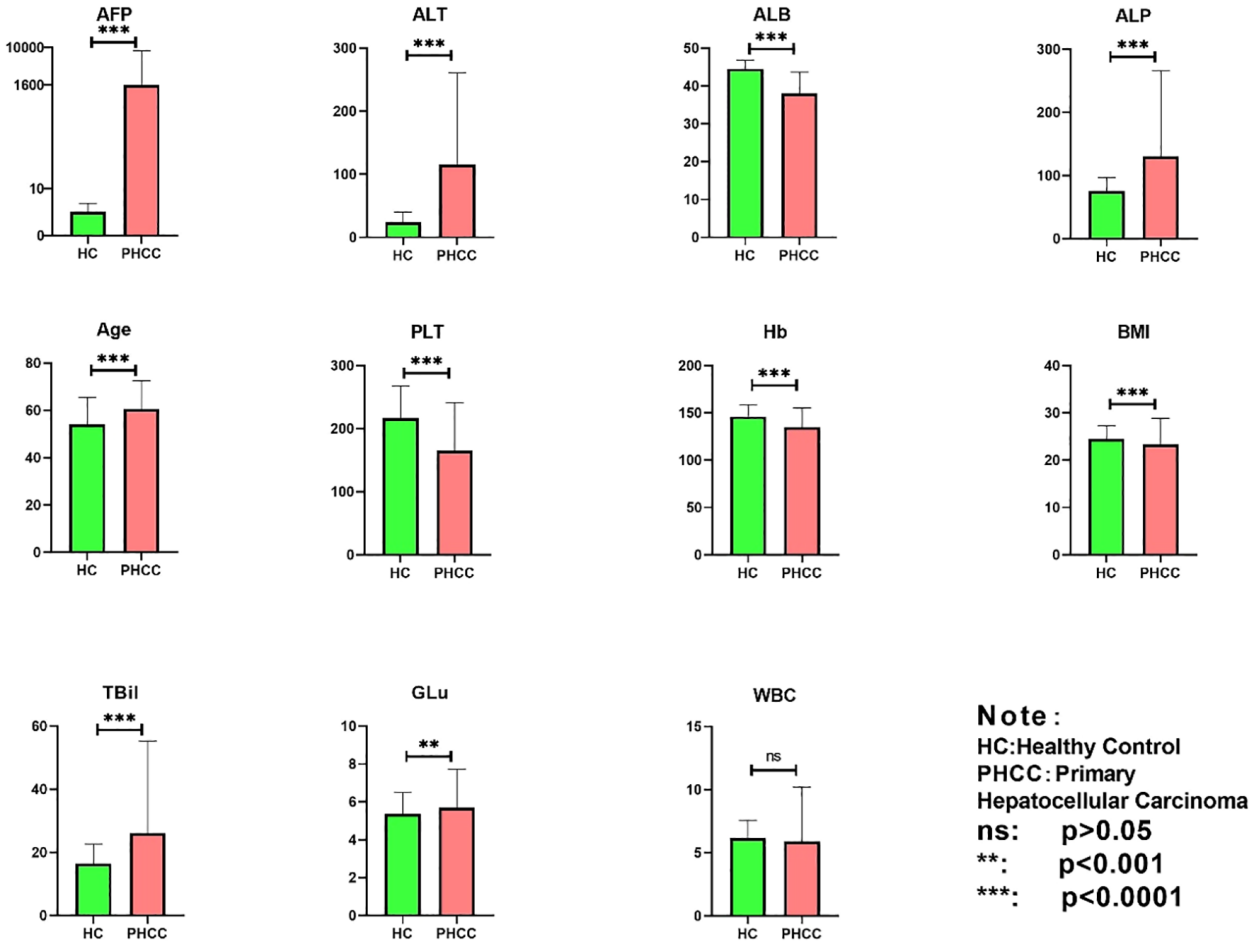
Statistical analysis of 11 characteristics in the PHC and healthy control groups. ns, indicates non-significant (p>0.05), **p<0.001, and ***p<0.0001.

A total of 2599 samples were included in this study, with 712 in the PHC group and 1887 in the HC group. The information of the demographics and blood indicator levels included 11 features, which were analyzed statistically using one-way ANOVA. The results are shown in **Figure 3**. Notably, no significant differences between the groups were observed in terms of WBC counts (P > 0.05). However, there were significant differences in the GLu, BMI, AFP, HB, PLT, ALB, TBil, ALT, and ALP values between the PHC and HC group (P< 0.05). Thus, the “WBC” characteristic lacks a significant reference value for distinguishing between the PHC and HC groups, while the remaining 10 features may be independent factors affecting the diagnosis of PHC.

The datasets for the PHC and HC group showed an imbalance in the current classification procedure. To address this issue, a random downsampling algorithm was adopted to acquire 712 HC samples. The leave-out method was then used to divide the dataset into training and test sets at an 8:2 ratio, respectively. To maximize the predictive potential of machine learning, mainstream ensemble learning models, namely, RF, LightGBM, Xgboost, and Catboost, were used for classification of the PHC and HC group, using all 11 features. Furthermore, for the purpose of baseline comparison, this paper also employs three classic machine learning methods - MLP, SVM, and KNN - to build models. A 50-fold cross-validation technique was used to assess the diagnostic performance of the ensemble learning models. This involved splitting the dataset into 50 equally sized folds. The model underwent 50 rounds of training and validation, using a different fold for validation in each iteration and the remaining forty-nine folds for training. This iterative process encompassed all folds, and an estimate of the overall performance of the model was assessed by averaging the results. This approach ensured a more reliable estimate of the performance of the model than the use of a single train-test split, which could introduce bias according to the specific data selected, and thus helped mitigate overfitting concerns.

To validate the diagnostic performance of the 11 signatures, a set of ensemble learning algorithms (RF, LightGBM, Xgboost, and Catboost) and machine learning algorithms (MLP, SVM, and KNN) was used to assess the model. The model was validated by comparing the diagnostic efficiency, accuracy, and precision of the different algorithms (**Table 1**). This showed that the Catboost, XgBoost, and RF models were effective in distinguishing between the PHC and HC groups, with all showing an accuracy above 96%. The Catboost model was especially noteworthy, with an accuracy of 96.40 ± 0.32% and ranking top in precision, recall, and the F1-score. In comparison to ensemble learning models, the performance of classical machine learning models is not particularly outstanding. These findings demonstrate the effectiveness of ensemble learning algorithms for the precise differentiation between patients with PHC and HC group.

**Table 1:** Machine learning model ratings.

Model	Accuracy	Precision	Recall	F1-score
Catboost Xgboost RF LightGBM MLP SVM KNN	96.40 ± 0.32 96.33 ± 0.47 96.08 ± 0.41 95.80 ± 0.41 95.13 ± 0.39 94.35 ± 0.58 91.31 ± 0.75	96.61 ± 1.21 96.21 ± 1.32 95.85 ± 1.59 95.61 ± 1.52 94.83 ± 1.31 95.03 ± 1.56 93.98 ± 4.55	94.40 ± 4.76 94.57 ± 4.15 94.31 ± 4.17 93.85 ± 4.56 92.95 ± 5.15 90.81 ± 8.15 84.73 ± 14.95	95.41 ± 2.20 95.34 ± 2.27 95.03 ± 2.38 94.67 ± 2.56 93.81 ± 2.92 92.62 ± 3.71 88.00 ± 6.44

**Figure 4:**
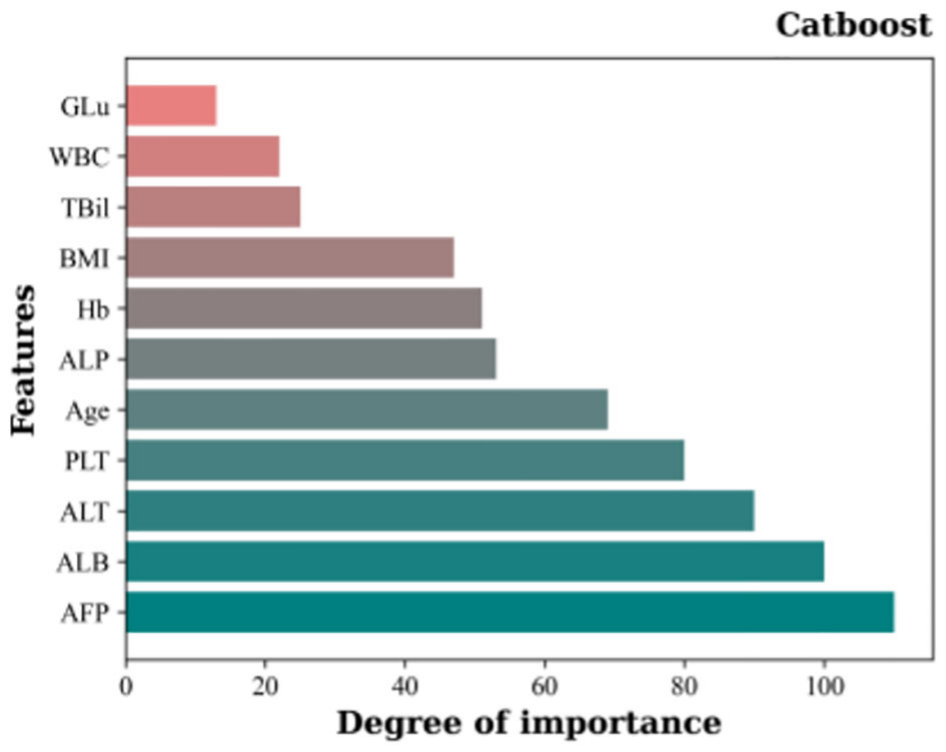
Importance rankings of 11 features based on Catboost and the RFE feature selection algorithm..

Using the top-performing classification model, Catboost, the RFE algorithm was used for feature selection using 10 random samples for classification. The results showing the respective importance of the different features are shown in **Figure 4**. Notably, all models identified AFP as the feature with the highest importance, followed by, in ranking order, ALB, ALT, PLT, age, ALP, Hb, BMI, TBil, WBC, and GLu.

**Figure 5:**
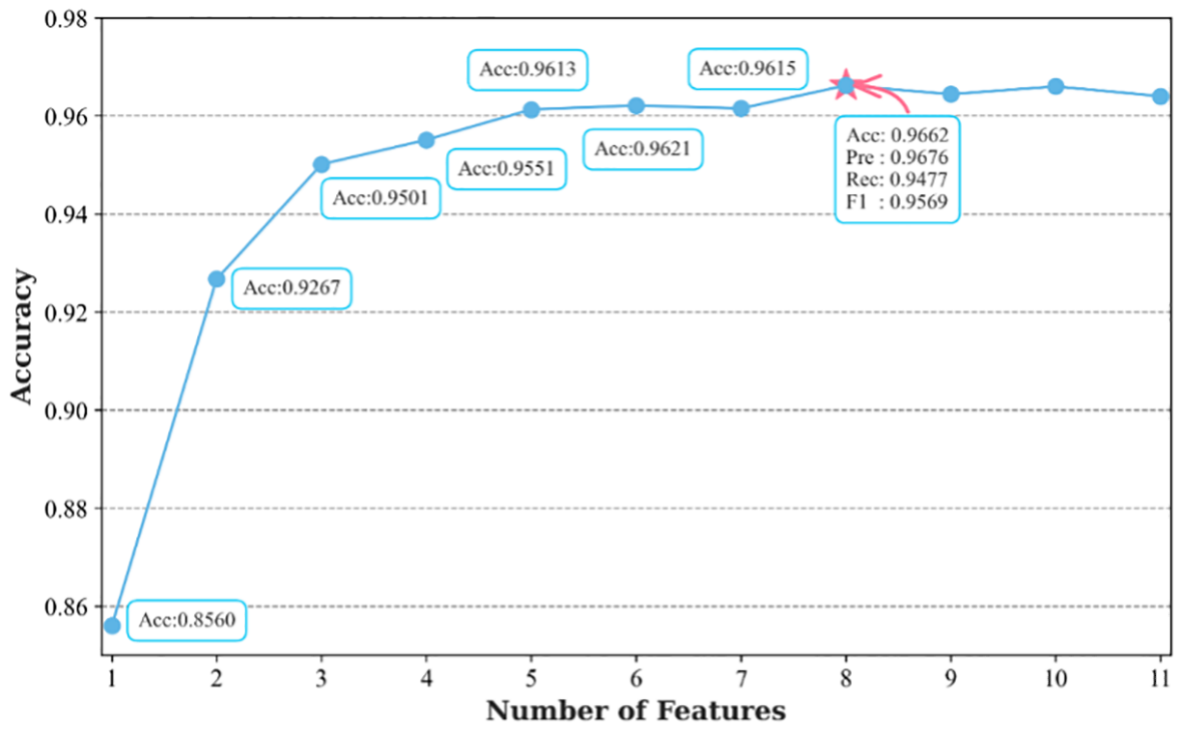
Classification performance after addition of the ranked features.

**Figure 6:**
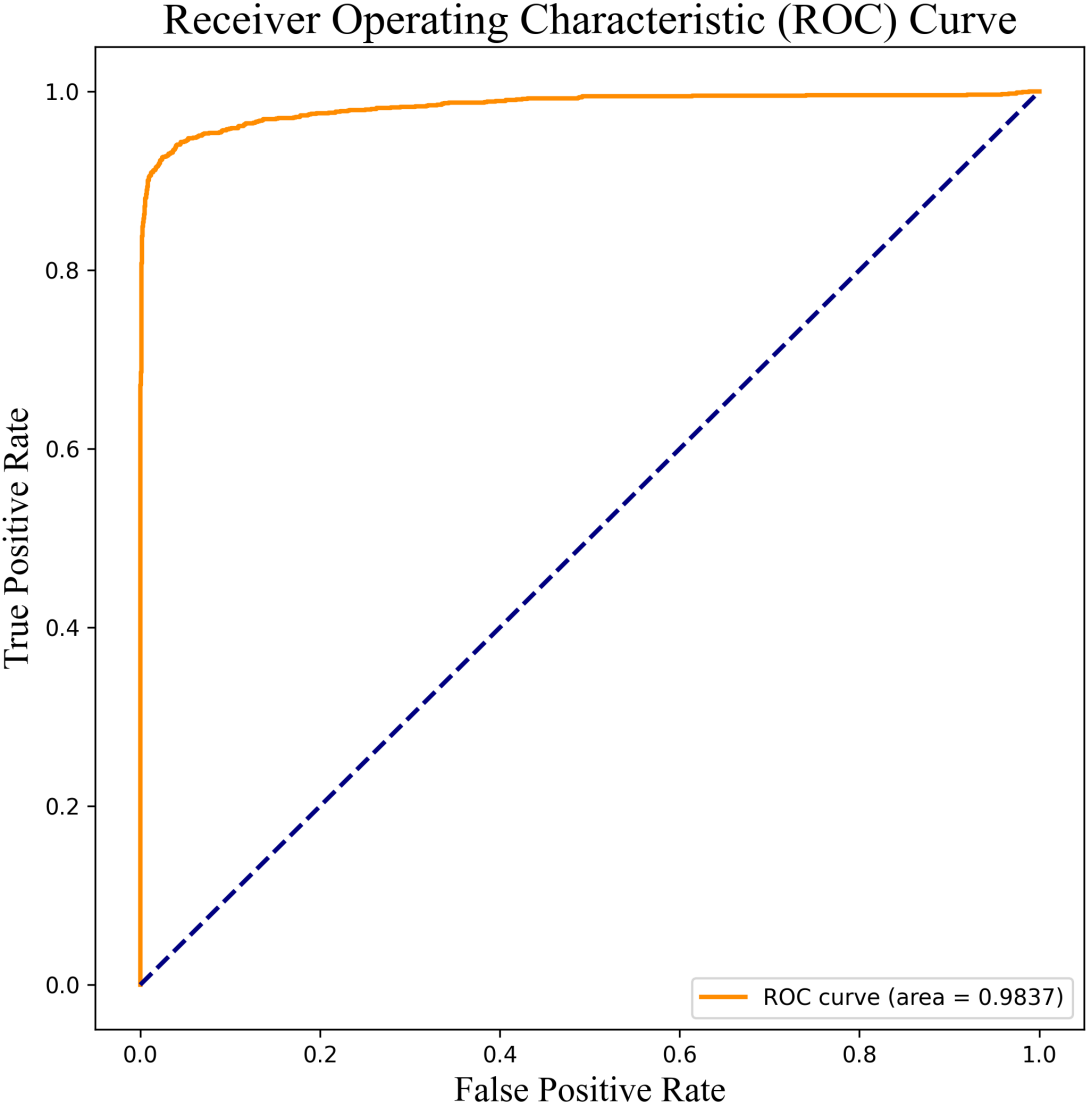
ROC curve of the optimal feature subset containing eight features.

The classification task involved the sequential addition of features according to the rankings shown in **Figure 4**, with incremental incorporation of individual features into each classification run until all features were included in the classifier. The results are shown in **Figure 5**. Initially, the model achieved 85.60% accuracy when only AFP was used. However, after the inclusion of ALB, ALT, and PLT, the accuracy rose significantly to 95.01%. Notably, the accuracy of the model increased incrementally on the addition of the features, although the accuracy was observed to decrease after the incorporation of TBil, WBC, and GLu. **Figure 5** illustrates the incorporation of the top 8 features based on their importance to yield the highest accuracy value, namely, 96.62%. This surpassed the 96.40% accuracy obtained using Catboost as the classification model, while also demonstrating higher precision, recall, and F1-score values. At the same time, we have plotted the receiver operating characteristic (ROC) curve for the optimal feature subset containing eight features, as shown in **Figure 6**. This curve visually demonstrates the performance of the model, and its high AUC (Area under the Curve of ROC) value convincingly proves that through the method of ensemble learning, we have successfully built a relatively accurate predictive model. This optimal subset of features, namely, AFP, ALB, ALT, PLT, age, ALP, Hb, and BMI, indicates their significant roles in the diagnosis of PHC and suggests their potential value as biomarkers in PHC classification.

## 5 Discussion

This study established a model for the diagnosis of PHC through the collection and processing of clinical data, using ensemble learning techniques. The key findings include the initial use of Catboost and the RFE algorithm to identify an optimal subset including eight features, which showed a classification accuracy of 96.62% in distinguishing between the PHC and HC group. Additionally, ensemble learning also identified critical hematological indicators, AFP, ALB, ALT, PLT, ALP, and Hb, together with two general clinical indicators, age and BMI. These findings present valuable insights for the preliminary screening for PHC and its subsequent diagnosis.

### 5.1 Ensemble learning was effective for PHC diagnosis

In recent years, preliminary detection of PHC has largely relied on US and AFP. However, the specificity and sensitivity of these methods remain suboptimal. To address this issue, many studies have focused on extracting features from imaging data to construct machine learning models. Recent literature reviews have shown the emergence of various US-based algorithms designed to differentiate between benign and malignant focal liver lesions (FLLs). These algorithms have demonstrated accuracy levels comparable to those of radiologists (27). Yang et al. designed a deep convolutional neural network (DCNN-US) to aid radiologists in distinguishing between benign and malignant FLLs using US. The model exhibited promising performance in a validation cohort, with a classification accuracy of 84.7%. However, it was found that the accuracy of the DCNN-US model was slightly inferior to that of enhanced MRI, which showed an accuracy of 87.9% (28). AI utilizing contrast-enhanced ultrasound (CEUS) was found to achieve a notable classification accuracy of 91% in distinguishing between benign and malignant FLLs, surpassing the performance of radiologists (29). However, when the model was compared to US, CT, and MRI, both CT and MRI showed higher resolution and superior contrast enhancement capabilities. Thus, machine learning models based on CT and MRI tend to yield higher classification accuracy (30–32). Despite this advantage, enhanced CT and MRI examinations are commonly used as definitive diagnostic modalities rather than for the preliminary screening for PHC.

As described above, while machine learning significantly reduces human influence and demonstrates a high accuracy in image recognition for the assessment of imaging test outcomes, the need to perform imaging tests such as US, CT, and MRI for every PHC diagnosis is a time-consuming process requiring experienced radiologists. Moreover, the unequal distribution of healthcare resources across geographical regions leads to challenges in making prompt and effective clinical decisions. Furthermore, not everyone can undergo enhanced CT or MRI due to potential allergic reactions to contrast agents, which can adversely affect kidney function. In contrast, screening methods based on serological data offer advantages such as cost-effectiveness, convenient sampling, and specific test result values that easily be integrated into algorithms. Additionally, these methods allow the combination of multiple indicators to enhance the accuracy of the test, the reason for the use of serological data in the present study.

The accumulation of medical data, including big data, and advances in ensemble learning methods invite their integration for the establishment of efficient models for the diagnosis of PHC. Johnson et al. (18) conducted a case-control study involving 331 patients with HCC and 339 patients with chronic liver disease (CLD) from a UK center. The study resulted in the development of a GALAD model, incorporating gender, age, the alpha-fetoprotein heteroplasmy ratio (AFP-L3), AFP, and Des-gamma-carboxy prothrombin (DCP), for the early diagnosis of PHC. The performance of the model was validated further in the UK, Germany, and Japan (33, 34). Liu et al. (35) assessed the performance of the GALAD model in the Chinese population and established a revised version, the GALAD-C model, using the same five variables as GALAD. They also introduced the GAAP model that incorporated gender, age, AFP, and protein induced by vitamin K absence or antagonist-II (PIVKA-II). In the test set, the GAAP model was found to have a classification accuracy of 89% in distinguishing between patients with HCC and HC, although this did not apply to HCC patients infected with HBV. Notably, the GALAD model, which was developed based on cohorts in the UK, was found to have higher pooled sensitivity and accuracy for the identification of HCC in Western countries compared to East Asian countries (36). As described above, the etiology of PHC is not the same in all parts of the world, with HBV infection being the predominant cause in China, HCV infection in Japan, and alcoholic hepatitis and non-alcoholic fatty liver disease (NAFLD) predominating in Western countries. To address this issue, Yang et al. (37) devised the ASAP model specifically within the context of an HBV-infected population. This model incorporated four independent risk factors as predictive variables, namely, patient’s age, sex, AFP and PIVKA-II levels. In the test set, this model exhibited a sensitivity of 87.8% and a specificity of 81.0%. However, the model was not validated further, necessitating additional investigation into its performance. Numerous studies have demonstrated the superiority of machine learning models over traditional approaches in the identification and prediction of PHC. For instance, Audureau et al. (38) and Ioannou et al. (39) used machine learning algorithms to predict HCC risk in patients with cirrhosis caused by HCV infection, both concluding that the algorithms enhanced the accuracy of prediction. Wong et al. (40), utilizing five machine learning methods, developed the HCC ridge score (HCC-RS), which was found to surpass the predictive performance of existing HCC risk scores. Comparative information on some of the above mentioned PHC prediction models can be found in **Table 2**. In the present study, an ensemble learning approach yielded a prediction model with robust predictive ability, achieving an accuracy of 96.62% and an AUC of 0.9837. This model outperformed the already reported models listed in **Table 2**, indicative of its superior predictive efficacy. Moreover, the ensemble learning model has potentially broad applications as it uses fundamental and easily accessible clinical indicators. This model can not only be applied to the HBV-infected population but can also analyze and predict biological indicators of individuals undergoing health checkups. Such versatility would reduce the burden on both patients and examiners.

**Table 2:** The comparison of PHC prediction models.

	GALAD (18)	GALAD-C (35)	GAAP (35)	ASAP (37)	HCC-RS (40)
**Algorithm**	Logistic regression	Logistic regression	Logistic regression	Logistic regression	Ridge regression
**Components**	Gender, Age, AFP-L3, AFP, DCP	Gender, Age, AFP-L3, AFP, DCP	Gender, Age, AFP, PIVKA-II	Age, Sex, AFP, PIVKA-II	Liver biochemistries, hematological and virological parameters
**Accuracy**	91.00	85.30	89.00	86.30	95.60
**Sensitivity**	93.00	82.60	87.20	87.80	52.00
**Specificity**	89.00	98.00	98.00	81.00	90.00
**AUC**	0.966	0.982	0.979	0.902	0.842

### 5.2 Ensemble learning identifies PHC biomarkers

The present study included 11 features, leading to the ultimate selection of AFP, ALB, ALT, PLT, age, ALP, Hb, and BMI as the optimal subset of features after ensemble learning and feature selection. AFP was originally discovered in human fetal serum by Bergstrand and Czar (41) and represents the first serological marker used in the clinical diagnosis of HCC. It is also the most widely used adjunctive test clinically employed for the screening, diagnosis, and evaluation of PHC (42). Recent studies have reported that patients with certain benign liver diseases (such as active hepatitis and cirrhosis) might also exhibit elevated levels of AFP in their blood. Additionally, malignancies such as gonadal and gastrointestinal tumors can also contribute to increased AFP levels (43, 44). However, despite being discovered over 60 years ago, AFP remains widely used today. Notably, in China, AFP demonstrates a good detection rate due to variations in causal factors (45). Additional reasons are differences in treatment modalities and the awareness of citizens’ health concerns. In China, patients are often diagnosed when PHC is at an advanced stage, presenting with noticeable symptoms and signs, when AFP levels are known to be elevated especially during the middle and advanced stages of PHC. Additionally, as indicated in **Figure 5**, it is apparent that the use of AFP only has an accuracy of only 85.60%. This underscores the issue that the use of single tumor markers for classification is less accurate. Thus, the present study included additional features to enhance the classification accuracy.

ALB, ALT, and PLT, together with AFP, were found to be the most significant features. Notably, significant improvements in accuracy were observed when these three features were included in the classifier. ALB and ALT are frequently used as clinical indicators of liver function, where ALB mirrors the level of synthesis in the liver and ALT indicates the extent of parenchymal damage. Hypoalbuminemia, prevalent in cirrhosis, is associated with decreased survival rates (46). In contrast, most patients with PHC have associated CLD, which progresses from liver disease to cirrhosis and finally to PHC. Currently, the commonly used clinical tools for assessing liver function in PHC are the Child-Pugh class assessment and the albumin-bilirubin (ALBI) classification, both of which incorporate ALB. ALT levels are positively associated with the degree of liver injury and serve as an additional predictive factor for PHC progression. Previous studies (47–49) have demonstrated that sustained abnormalities in serum ALT levels are an independent risk factor for PHC, consistent with the findings of the present study. Furthermore, Kim et al. (50) confirmed that rapid normalization of ALT levels through continuous antiviral therapy may mitigate the risk of HCC in individuals with HBV-related cirrhosis. Notably, approximately 90% of confirmed PHC cases showed elevated levels of both ALT and ALB in a previous case-control study (51). It has been a long-standing and widely accepted view that abnormal changes in PLT are associated with tumor progression and resistance to chemotherapeutic agents through multiple mechanisms (52). Cirrhosis eventually leads to portal hypertension and hypersplenism and causes thrombocytopenia. Thrombocytopenia has been identified as a key risk factor for the development of cirrhosis and hepatocarcinogenesis in CLD (53, 54). Recent studies on PLT level in PHC patients have focused on the relationships between PLT count or morphological changes and PLT-related ratios and their prognostic predictions. For instance, it has been found that PLT acts as a simple, cost-effective, and efficient predictor of survival in PHC patients (55). Additionally, the PLT level has been shown to be a predictor of recurrence in patients with PHC after surgical resection (56). Fan et al. (57) not only confirmed the role of PLT in the tumor microenvironment of PHC but also showed that PLT count were proportional to tumor size. Many studies have shown that anti-PLT therapy reduces both PLT activity and CD8+ T-lymphocyte infiltration, preventing liver fibrosis and the development of PHC (58–60). These findings are consistent with the results obtained in the present study, confirming that PLT is a potential marker for PHC.

Although the inclusion of age, ALP, Hb, and BMI did not notably enhance the classification accuracy compared to the initial four features, the classifier achieved its peak accuracy of 96.62% with the selected eight features. Thus, these eight features were designated the optimal feature subset. Age and BMI represent general clinical information. **Figure 1** shows that the patients with PHC were predominantly concentrated in the middle and older age groups, indicating an increased likelihood of developing PHC with advancing age. This underscores the role of age as a notable risk factor in the development of PHC. It has also been found that obesity is a risk factor for PHC (61–63), and current data indicate a rapid increase in the incidence of NAFLD-associated HCC throughout the world. The BMI value, which is closely related to the total amount of body fat, is a commonly used measure of how fat or thin the body is and whether it is healthy or not, and was therefore included in our study, finding that BMI was a potential marker for PHC. ALP is elevated in metabolic disorders, inflammation, and tumorigenesis (64). It is mainly used clinically for the diagnosis and differential diagnosis of diseases of the skeletal and hepatobiliary systems. Many researchers consider ALP to be an independent prognostic factor for PHC after partial hepatectomy (65, 66). Hb, which is commonly used clinically for the evaluation of anemia, was introduced as a new potential marker to predict the clinical course and outcomes of patients with various cancer types in a meta-analysis, which concluded that cancer patients with lower Hb levels were more likely to experience disease progression and recurrence (67). While these features may not represent individual markers for PHC screening, the diagnostic efficacy of the models in the present study was found to markedly improve after their inclusion.

The remaining TBil, WBC, and GLu factors were ranked as the least important, and the accuracy of the model decreased after their addition to the classifier. TBil is an indicator of bilirubin metabolism, and significantly elevated TBil levels are associated with jaundice, which is often observed in moderately advanced PHC and in 5−44% of PHC patients (68). However, it is important to note that jaundice is not exclusive to PHC. Typically, TBil is combined with ALB to generate an ALBI score, which is used as a tool for the assessment of liver function and prognostic prediction in patients with PHC (69). WBC count, as a central component of the body’s defense system, typically has its fluctuations closely related to the presence of inflammation or infection. However, in the early stages of PHC, infection is not a primary or significant clinical manifestation. While we have observed that PHC patients sometimes experience hypersplenism, which may lead to a decrease in WBC count, this change is not specific to PHC, as various other diseases can also cause similar phenomena. Additionally, through statistical analysis, we have found that WBC count does not exhibit statistical significance. There are a growing number of epidemiological studies that suggest that diabetes may increase cancer risk but the association between type 2 diabetes mellitus (T2DM) and PHC has focused on NAFLD (70–72), which does not confirm the diagnostic value of GLu for PHC. This argument supports the conclusion that TBil, WBC, and GLu do not have sufficient discriminatory power in the classification and diagnosis of PHC.

### 5.3 Significance of the study

After a thorough investigation into the application of ensemble learning algorithms in the prediction and diagnosis of PHC, we have established its significant and indispensable importance in the field. This groundbreaking discovery has furnished clinicians with a robust auxiliary tool, significantly enhancing their ability to make precise and reliable diagnostic assessments. With the incremental integration of ensemble learning algorithms in PHC prediction and diagnosis, existing screening techniques have undergone substantial optimization and advancement. Looking forward, we anticipate that ensemble learning algorithms, grounded in serological and demographic indicators, will assume an increasingly pivotal role in advancing medical prediction and diagnostic technologies, enabling the primary detection of PHC signs. This will afford patients an earlier window for therapeutic intervention, improving the timeliness and efficacy of treatment, and ultimately leading to a significant increase in the success rate of PHC treatment.

### 5.4 Limitations

The results obtained using ensemble learning to analyze clinical data provide a method for the diagnosis and risk assessment of PHC, thus potentially introducing innovative avenues for PHC diagnosis and screening. Nevertheless, the study has several limitations. First, the data were sourced from a single medical center, potentially introducing the problems of bias and unbalanced sample distribution. Thus, the collection of additional data using multi-center studies would enhance the generalizability of the findings. Secondly, the included PHC patients lacked clinical staging information, which is crucial for assessing the disease status, treatment strategies, and predicting prognosis of PHC patients. Therefore, we need to supplement and improve the patients’ clinical staging information in our future work. Thirdly, due to the lack of data from patients with hepatitis and cirrhosis in the control group, we have been unable to delve deeply into the differences between hepatitis, cirrhosis, and PHC. Given the limited data we have collected on other similar PHC diseases, the current validation of our method’s effectiveness remains inadequate. Therefore, we will continue to work on addressing this issue in order to better verify and refine our method. Moreover, external validation of the model and prospective studies to verify its efficacy, together with imaging data, are needed in future research.

## 6 Conclusion

PHC is a common malignancy that presents significant health risks. In the era of medical big data, the harnessing of hidden information through the use of algorithmic tools such as ensemble learning has gained prominence. The present study aimed to derive quantifiable and precise biological markers using ensemble learning techniques, achieving a peak accuracy of 96.62% in the detection of PHC. Feature selection and ranking were also performed to identify the optimal subset of features delivering the highest classification accuracy. This process identified a subset of biomarkers with high diagnostic efficacy, specifically, AFP, ALB, ALT, PLT, age, ALP, Hb, and BMI. These findings underscore the importance of employing ensemble learning algorithms in the prediction and diagnosis of PHC. The results have the potential to assist clinicians in decision-making, thereby advancing techniques used for PHC screening. During physical examinations or routine blood tests, early warnings can be issued to prompt further investigations, thus reducing the missed diagnosis rate. This will provide patients with an earlier opportunity for treatment, significantly improving the success rate of treatment, and injecting new vitality into the prevention and control of PHC.

## Data availability statement

The original contributions presented in the study are included in the article/**Supplementary Material**. Further inquiries can be directed to the corresponding authors.

## Ethics statement

The studies involving humans were approved by Ethics Committee of Jinhua Hospital Affiliated to Zhejiang University (protocol code 2021-252-001 and 1 November 2021). The studies were conducted in accordance with the local legislation and institutional requirements. The participants provided their written informed consent to participate in this study.

## Author contributions

MW: Conceptualization, Data curation, Writing – original draft. BZ: Funding acquisition, Resources, Software, Writing – original draft. GL: Conceptualization, Formal analysis, Methodology, Supervision, Validation, Writing – review & editing. SY: Project administration, Supervision, Validation, Writing – review & editing.
