# Supplementary material for: Ensemble learning enhances the precision of preliminary detection of primary hepatocellular carcinoma based on serological and demographic indices
Source: Front Oncol. 2024 Jun 17;14:1397505. doi: 10.3389/fonc.2024.1397505 (PMC11215019; doi:10.3389/fonc.2024.1397505)
Supplement: Supplementary file 2 [file Table_2.docx]

Supplementary Material

**Ensemble Learning Enhances the Precision of Preliminary Detection of Primary Hepatocellular Carcinoma Based on Serological and Demographic Indices**

**Mengxia Wang^1†^, Bo Zhuang^2†^, Shian Yu^2*^ and Gang Li^3*^**

*** Correspondence:** Shian Yu: [ysa513@163.com](mailto:ysa513@163.com); Gang Li: ligangsdu@foxmail.com

# Hyperparameter Details of the ML models

The hyperparameters of each model used in the study are listed in the following table:

| Model | Parameter | Value |
| --- | --- | --- |
| RF | max_depth | None |
|  | n_estimators | 100 |
|  | criterion | gini |
|  | min_samples_leaf | 1 |
|  | min_samples_split | 2 |
| LightGBM | n_estimators  num_leaves | 100  31 |
|  | min_child_weight | 1e-3 |
|  | min_child_samples | 20 |
|  | learning_rate | 0.1 |
| Xgboost | max_depth  n_estimators | 6  100 |
|  | colsample_bytree | 1.0 |
|  | min_child_weight | 1 |
|  | learning_rate | 0.3 |
| Catboost | iterations  depth | 1000  6 |
|  | loss_function | LogLoss |
|  | learning_rate | 0.03 |
| MLP | hidden_layer_sizes | (100,) |
|  | activation | relu |
|  | solver | adam |
|  | max_iter  learning_rate | 200  0.001 |
| KNN | n_neighbors | 5 |
|  | weights | uniform |
|  | leaf_size | 30 |
|  | metric | minkowski |
| SVM | kernel | rbf |
|  | degree  cache_size  decision_function_shape | 3  200  ovr |
